# Supplementary material for: Giant g-factors and fully spin-polarized states in metamorphic short-period InAsSb/InSb superlattices
Source: Nat Commun. 2022 Oct 10;13:5960. doi: 10.1038/s41467-022-33560-x (PMC9551044; doi:10.1038/s41467-022-33560-x)
Supplement: Supplementary file 1 — Supplementary Information [file 41467_2022_33560_MOESM1_ESM.pdf]

Supplementary Information: Giant g-factors and fully spin-polarized states in metamorphic short-period InAsSb/InSb superlattices

Yuxuan Jiang,[1,2,*] Maksim Ermolaev,[3,*] Gela Kipshidze,[3] Seongphill Moon,[2,4] Mykhaylo Ozerov,[2] Dmitry Smirnov,[2] Zhigang Jiang,[1,†] and Sergey Suchalkin[3,‡]

[1]*School of Physics, Georgia Institute of Technology, Atlanta, Georgia 30332, USA*

[2]*National High Magnetic Field Laboratory, Tallahassee, Florida 32310, USA*

[3]*Department of Electrical and Computer Engineering, Stony Brook University, Stony Brook, New York 11794, USA*

[4]*Department of Physics, Florida State University, Tallahassee, Florida 32306, USA*

---

* Y. J. and M. E. contributed equally to this work.

† zhigang.jiang@physics.gatech.edu

‡ sergey.suchalkin@stonybrook.edu

This file includes:
Supplementary Note 1. Additional data on sample 1444
Supplementary Note 2. Eight-band $k \cdot p$ calculation
Supplementary Note 3. Spin polarization calculation
Supplementary Note 4. Extended fitting between the $k \cdot p$ model and the four-band model
Supplementary Note 5. Effective g-factor error estimation
Supplementary Note 6. Origin of the band splitting
Supplementary Figure 1. Additional data on sample 1444
Supplementary Figure 2. Extended LL fitting in the short-period limit
Supplementary Figure 3. Extended LL fitting between two models
Supplementary Figure 4. Examples of raw spectra
Supplementary Figure 5. Error estimation in k.p fitting
Supplementary Table 1: Parameters for band structure calculations
Supplementary Table 2: Parameters for strain effect

SUPPLEMENTARY NOTE 1. ADDITIONAL DATA ON SAMPLE 1444

Superlattice (SL) sample 1444 has 213 periods; each period includes 3 nm of $InAs_{0.48}Sb_{0.52}$ and 1.69 nm of InSb. Supplementary Fig. 1 shows the false color plot of the relative transmission $T(B)/T(0)$ of the sample as a function of energy and magnetic field. A series of spectral dips (i.e., absorptions) can be identified, blueshifting with increasing magnetic field. The black dash lines represent the calculated Landau level (LL) transition energies at different magnetic fields using the $k \cdot p$ model. Excellent agreement between the calculation and the experiment is achieved with the same set of band parameters for samples 1445 and 1446. The first five low-lying transitions are numbered ($T_{2,\ldots,6}$) in sequence of their energies, consistent with that in Figs. 3(a) and 3(b) of the main text. The absence of the T_1 transition, a cyclotron resonance (CR) transition, indicates that the sample is more intrinsic than 1445 and 1446.

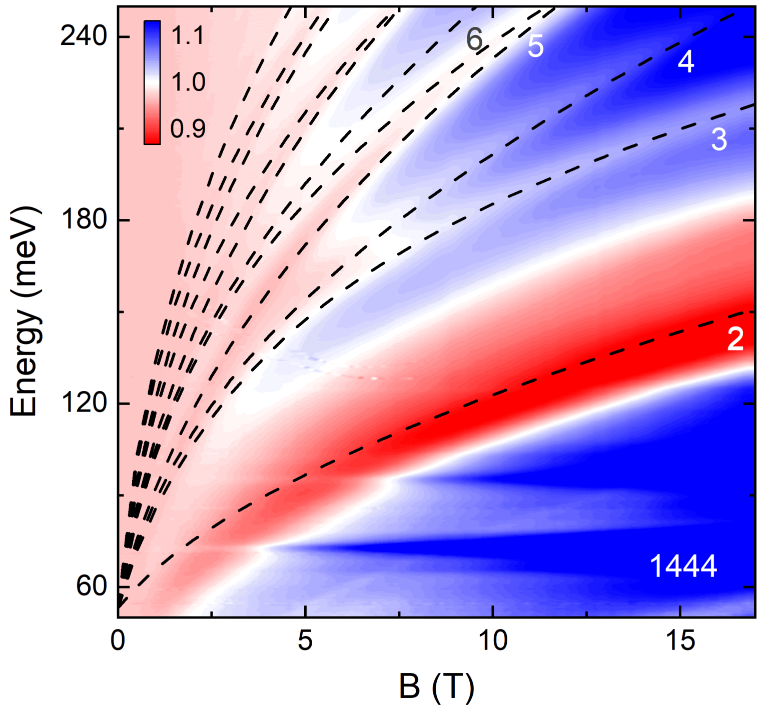

Supplementary Fig. 1. Additional data on sample 1444. False color map of magneto-absorption at different energies for sample 1444. The black dash lines represent the calculated LL transition energies at different magnetic fields. The first five low-lying transitions are numbered ($T_{2,\ldots,6}$) in sequence of their energies, consistent with that in Figs. 3(a) and 3(b) of the main text. The absence of the T_1 transition, a CR transition, indicates that the sample is more intrinsic than 1445 and 1446. All measurements are performed at 5 K.

SUPPLEMENTARY NOTE 2. EIGHT-BAND $k \cdot p$ CALCULATION

To correctly interpret the magneto-infrared spectroscopy data, we perform the $k \cdot p$ calculations [1–5]. We break down the total Hamiltonian into three parts

$$H = H_{k \cdot p} + H_s + H_Z,$$

which take into account the material band structure [4], the strain effect [5], and the Zeeman effect [3], respectively. The $H_{k \cdot p}$ Hamiltonian near Γ point for zinc-blende type crystals is

well documented. We employ the following basis

$$
\begin{aligned}
\phi_1 &= \left|\frac{1}{2},\frac{1}{2}\right\rangle = |S\uparrow\rangle\,, & \phi_2 &= \left|\frac{1}{2},-\frac{1}{2}\right\rangle = |S\downarrow\rangle\,,\\
\phi_3 &= \left|\frac{3}{2},\frac{3}{2}\right\rangle = \frac{1}{\sqrt{2}}\,|(X+iY)\uparrow\rangle\,, & \phi_4 &= \left|\frac{3}{2},\frac{1}{2}\right\rangle = \frac{i}{\sqrt{6}}\,|(X+iY)\downarrow -2Z\uparrow\rangle\,,\\
\phi_5 &= \left|\frac{3}{2},-\frac{1}{2}\right\rangle = \frac{1}{\sqrt{6}}\,|(X-iY)\uparrow +2Z\downarrow\rangle\,, & \phi_6 &= \left|\frac{3}{2},-\frac{3}{2}\right\rangle = \frac{i}{\sqrt{2}}\,|(X-iY)\downarrow\rangle\,,\\
\phi_7 &= \left|\frac{1}{2},\frac{1}{2}\right\rangle = \frac{1}{\sqrt{3}}\,|(X+iY)\downarrow +Z\uparrow\rangle\,, & \phi_8 &= \left|\frac{1}{2},-\frac{1}{2}\right\rangle = -\frac{i}{\sqrt{3}}\,|(X-iY)\uparrow -Z\downarrow\rangle\,,
\end{aligned}
$$

which corresponds to the electron spin up, electron spin down, heavy-hole spin up, light-hole spin up, light-hole spin down, heavy-hole spin down, split-off spin up, and split-off spin down states, respectively. The $H_{k\cdot p}$ Hamiltonian then takes the form of

$$
H_{k\cdot p} = \begin{bmatrix}
A & 0 & i\sqrt{3}V^\dagger & \sqrt{2}U & iV & 0 & iU & \sqrt{2}V\\
0 & A & 0 & -V^\dagger & i\sqrt{2}U & -\sqrt{3}V & i\sqrt{2}V^\dagger & -U\\
-i\sqrt{3}V & 0 & -(P+Q) & L & M & 0 & \frac{i}{\sqrt{2}}L & -i\sqrt{2}M\\
\sqrt{2}U & -V & L^\dagger & -(P-Q) & 0 & M & i\sqrt{2}Q & i\sqrt{\frac{3}{2}}L\\
-iV^\dagger & -i\sqrt{2}U & M^\dagger & 0 & -(P-Q) & -L & -i\sqrt{\frac{3}{2}}L^\dagger & i\sqrt{2}Q\\
0 & -\sqrt{3}V^\dagger & 0 & M^\dagger & -L^\dagger & -(P+Q) & -i\sqrt{2}M^\dagger & -\frac{i}{\sqrt{2}}L^\dagger\\
-iU & -i\sqrt{2}V & -\frac{i}{\sqrt{2}}L^\dagger & -i\sqrt{2}Q & i\sqrt{\frac{3}{2}}L & i\sqrt{2}M & -P-\Delta & 0\\
\sqrt{2}V^\dagger & -U & i\sqrt{2}M^\dagger & -i\sqrt{\frac{3}{2}}L^\dagger & -i\sqrt{2}Q & \frac{i}{\sqrt{2}}L & 0 & -P-\Delta
\end{bmatrix},
$$

where

$$
\begin{aligned}
A &= E_v + E_g + \boldsymbol{k}A_c\boldsymbol{k}, & Q &= \frac{\hbar^2}{2m_0}(k_x\gamma_2k_x + k_y\gamma_2k_y - 2k_z\gamma_2k_z),\\
P &= -E_v + \frac{\hbar^2}{2m_0}\boldsymbol{k}\gamma_1\boldsymbol{k}, & M &= -\frac{\sqrt{3}\hbar^2}{2m_0}[k_x\gamma_2k_x - k_y\gamma_2k_y - 2i\{k_x\gamma_3k_y\}],\\
L &= i\frac{\sqrt{3}\hbar^2}{m_0}\{k_-\gamma_3k_z\}, & U &= \frac{1}{\sqrt{3}}P_0k_z, \quad V = \frac{1}{\sqrt{6}}P_0k_-.
\end{aligned}
$$

Here, $\hbar$ is the reduced Planck constant, $\mathbf{k} = (k_x, k_y, k_z)$ is the wave vector, $k_\pm = k_x \pm ik_y$, $\{k_\alpha\gamma k_\beta\} = (k_\alpha\gamma k_\beta + k_\beta\gamma k_\alpha)/2$ where $\alpha, \beta = x, y, z$, E_v is the valence band offset, E_g is the band gap, Δ is the split-off band gap, γ_1,γ_2,γ_3 are the modified Luttinger parameters, m_0 is the free electron mass, and P_0 is related to the Kane energy E_p by $E_p = 2m_0P_0^2/\hbar^2$. Also, A_c is related to the electron effective mass m^* by $A_c = \hbar^2/2m^* - E_p(3E_g+2\Delta)/6m_0E_g(E_g+\Delta)$.

In our calculation, we define the SL growth direction as the z direction. While k_x and k_y are still good quantum numbers due to the in-plane translational symmetry, we need

to consider both the quantum confinement effect and the additional minibands from the thin film and periodic SL structure along the z direction. Therefore, we replace k_z with $k_z = -i\partial/\partial_z + k_\zeta$, where the momentum operator arises from the quantum confinement effect and k_ζ stands for the minibands caused by the periodic SL structure. Specifically, k_ζ takes a value between $-\pi/t$ and π/t, where t is the period of the SL. In the presence of a magnetic field (B), we focus on the LL spectrum at $k_\zeta = 0$ since this is the van Hove singularity point where dominant optical absorption occurs.

Next, we consider the strain effect, which plays an important role in semiconductor structures. The strain Hamiltonian H_s takes the following form

$$H_s = \begin{pmatrix} A_\epsilon & 0 & 0 & 0 & 0 & 0 & 0 & 0 \\ 0 & A_\epsilon & 0 & 0 & 0 & 0 & 0 & 0 \\ 0 & 0 & -P_\epsilon - Q_\epsilon & -L_\epsilon & -M_\epsilon & 0 & -i\frac{1}{\sqrt{2}}L_\epsilon & i\sqrt{2}M_\epsilon \\ 0 & 0 & -L_\epsilon^* & -P_\epsilon + Q_\epsilon & 0 & -M_\epsilon & i\sqrt{2}Q_\epsilon & -i\sqrt{\frac{3}{2}}L_\epsilon \\ 0 & 0 & -M_\epsilon^* & 0 & -P_\epsilon + Q_\epsilon & L_\epsilon & i\sqrt{\frac{3}{2}}L_\epsilon^* & i\sqrt{2}Q_\epsilon \\ 0 & 0 & 0 & -M_\epsilon^* & L_\epsilon^* & -P_\epsilon - Q_\epsilon & i\sqrt{2}M_\epsilon & i\frac{1}{\sqrt{2}}L_\epsilon^* \\ 0 & 0 & i\frac{1}{\sqrt{2}}L_\epsilon^* & -i\sqrt{2}Q_\epsilon & -i\sqrt{\frac{3}{2}}L_\epsilon & -i\sqrt{2}M_\epsilon & -P_\epsilon & 0 \\ 0 & 0 & -i\sqrt{2}M_\epsilon^* & i\sqrt{\frac{3}{2}}L_\epsilon^* & -i\sqrt{2}Q_\epsilon & -i\frac{1}{\sqrt{2}}L_\epsilon & 0 & -P_\epsilon \end{pmatrix},$$

where,

$$A_\epsilon = a_c(\epsilon_{xx} + \epsilon_{yy} + \epsilon_{zz}), \quad P_\epsilon = -a_v(\epsilon_{xx} + \epsilon_{yy} + \epsilon_{zz}), \quad L_\epsilon = id(\epsilon_{xz} - i\epsilon_{yz}),$$
$$Q_\epsilon = -\frac{b}{2}(\epsilon_{xx} + \epsilon_{yy} - 2\epsilon_{zz}), \quad M_\epsilon = -\frac{\sqrt{3}}{2}b(\epsilon_{xx} - \epsilon_{yy}) + i\frac{2\sqrt{3}}{3}d\epsilon_{xy}.$$

Here, a_c, a_v, b, and d are deformation potentials. We apply the pseudomorphic approximation by assuming the lattice constants in the core structures throughout the SL pinned to the lattice constants of the virtual substrate. Then, the strain tensor reads

$$\epsilon_{xx} = \epsilon_{yy} = \frac{a_0 - a}{a}, \quad \epsilon_{zz} = -\frac{2C_{12}}{C_{11}}\epsilon_{xx}, \quad \epsilon_{ij} = 0 \quad \text{for} \quad i \neq j,$$

where a_0 and a are the pinned and original lattice constants, respectively, and C_{11} and C_{12} are both the stiffness constants.

In the presence of a magnetic field, we also include the Zeeman effect using

$$H_Z = \frac{\hbar^2}{m_0 l_c^2} \begin{pmatrix} \frac{1}{2} & 0 & 0 & 0 & 0 & 0 & 0 & 0 \\ 0 & -\frac{1}{2} & 0 & 0 & 0 & 0 & 0 & 0 \\ 0 & 0 & -\frac{3}{2}\kappa & 0 & 0 & 0 & 0 & 0 \\ 0 & 0 & 0 & -\frac{1}{2}\kappa & 0 & 0 & i\sqrt{\frac{1}{2}}(\kappa+1) & 0 \\ 0 & 0 & 0 & 0 & \frac{1}{2}\kappa & 0 & 0 & -i\sqrt{\frac{1}{2}}(\kappa+1) \\ 0 & 0 & 0 & 0 & 0 & \frac{3}{2}\kappa & 0 & 0 \\ 0 & 0 & 0 & -i\sqrt{\frac{1}{2}}(\kappa+1) & 0 & 0 & -(\kappa+\frac{1}{2}) & 0 \\ 0 & 0 & 0 & 0 & i\sqrt{\frac{1}{2}}(\kappa+1) & 0 & 0 & \kappa+\frac{1}{2} \end{pmatrix},$$

where $\kappa = \gamma_3 + \frac{2}{3}\gamma_2 - \frac{1}{3}\gamma_1 - \frac{2}{3}$ [3], and l_c is the magnetic length.

Supplementary Tab. 1 summarizes the parameters used in our eight-band calculations, and Supplementary Tab. 2 summarizes the strain parameters. The parameters for InSb in Supplementary Tabs. 1 and 2 are taken from Ref. [6]. For 52% Sb alloy, we use the interpolation scheme recommended in Ref. [6] while leaving E_p and E_v as fitting parameters to match the experimental data [7, 8].

Supplementary Tab. 1: Parameters for band structure calculations.

Sb	E_v (eV)	E_g (eV)	Δ (eV)	γ_1	γ_2	γ_3	E_p (eV)	κ
52%	1.80	0.115	0.313	2.26	-0.578	0.278	18.0	-1.53
100%	2.017	0.235	0.81	1.75	-1.025	-0.025	23.3	-1.96

Supplementary Tab. 2: Parameters for strain effect

Sb	a (Å)	C_{11} (GPa)	C_{12} (GPa)	a_c (eV)	a_v (eV)	b (eV)
52%	6.268	755.8	411.5	-6.05	-0.67	-1.9
100%	6.469	684.7	373.5	-6.94	-0.36	-2.0

In all calculations, we use a piecewise function to describe the change of band parameters across the interface, and for simplicity, we take the axial approximation by replacing γ_2 and γ_3 with their average. In addition, we have rescaled the Kane energy E_p so that $A_c = 0$ to avoid spurious solutions [9].

Lastly, it is worth noting that for the split-off band gap Δ in $InAs_{0.48}Sb_{0.52}$, we consider the bowing effect [6] with a positive bowing parameter. Though both a positive [7, 10, 11]

and negative [12–14] bowing parameter have been reported in the literature, we choose a positive value to be self-consistent with that in Ref. [11] measured in similar SL samples. We also find that because the split-off band is far away in energy and only affects the band structure through perturbation, the choice of bowing parameter for Δ has a negligible effect on the model fitting in the main text.

SUPPLEMENTARY NOTE 3. SPIN POLARIZATION CALCULATION

Based on the above basis functions, we can calculate the spin polarization of the mth LL by summing up the corresponding spin components in the wavefunction spinor

$$P_{m,\uparrow} = \sum_{k=1,3,4,7} |f_k^m(z)|^2, \qquad P_{m,\downarrow} = 1 - P_{m,\uparrow},$$

where $\uparrow\downarrow$ denotes the spin directions, and f_k^m is the coefficient of the kth component in the eigenstates of the mth LL.

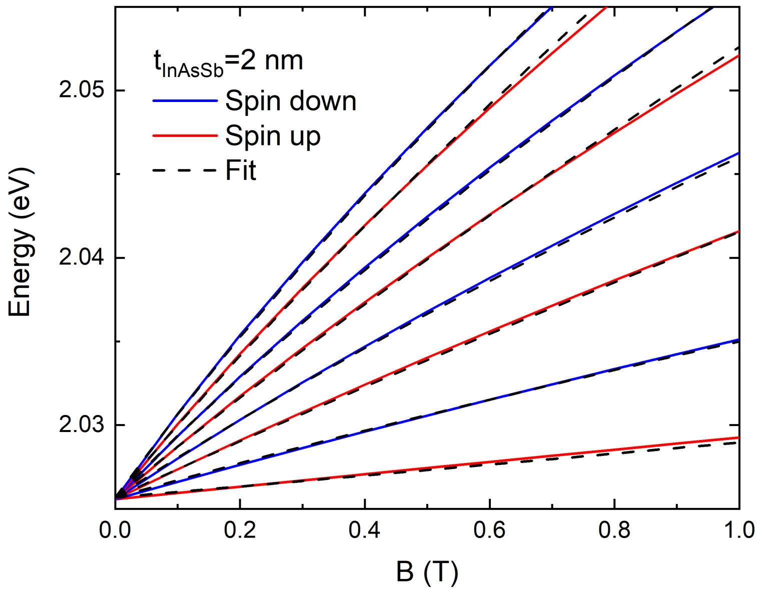

Supplementary Fig. 2. Additional LL fitting in the short-period limit. The calculated low-lying LLs from the $k \cdot p$ model (solid lines) for $InAs_{0.48}Sb_{0.52}/InSb$ (2-nm/1.12-nm) SL and fit with the four-band model (dash lines) at low magnetic fields. The red and blue colors correspond to the spin up and down LLs, respectively.

SUPPLEMENTARY NOTE 4. EXTENDED FITTING BETWEEN THE $k \cdot p$ MODEL AND THE FOUR-BAND MODEL

In Fig. 5(a,c) of the main text, we fit the low-field $k \cdot p$ LLs with the effective Hamiltonian of the four-band model for two ultra-narrow band gap SLs, and good agreement between the two models is achieved. In Supplementary Fig. 2, we show an additional fitting for the case of $InAs_{0.48}Sb_{0.52}/InSb$ (2-nm/1.12-nm) SL, where the band gap is relatively large $E_g \approx 103$ meV and in the ultrashort period limit. Great agreement between the two models is also evidenced.

To better understand the origin of the LL (spin) splitting in InAsSb/InSb SLs, we extend the fitting to a moderate high magnetic field. We consider a SL in the normal regime with a period of 3-nm/1.69-nm (sample 1444) as an example. Supplementary Fig. 3 shows the comparison between the $k \cdot p$ results and the four-band model with two different splitting mechanisms. In Supplementary Fig. 3(a), the splitting is solely due to the parabolic band (PB) component M_1, while in Supplementary Fig. 3(b), it is solely due to the Zeeman effect

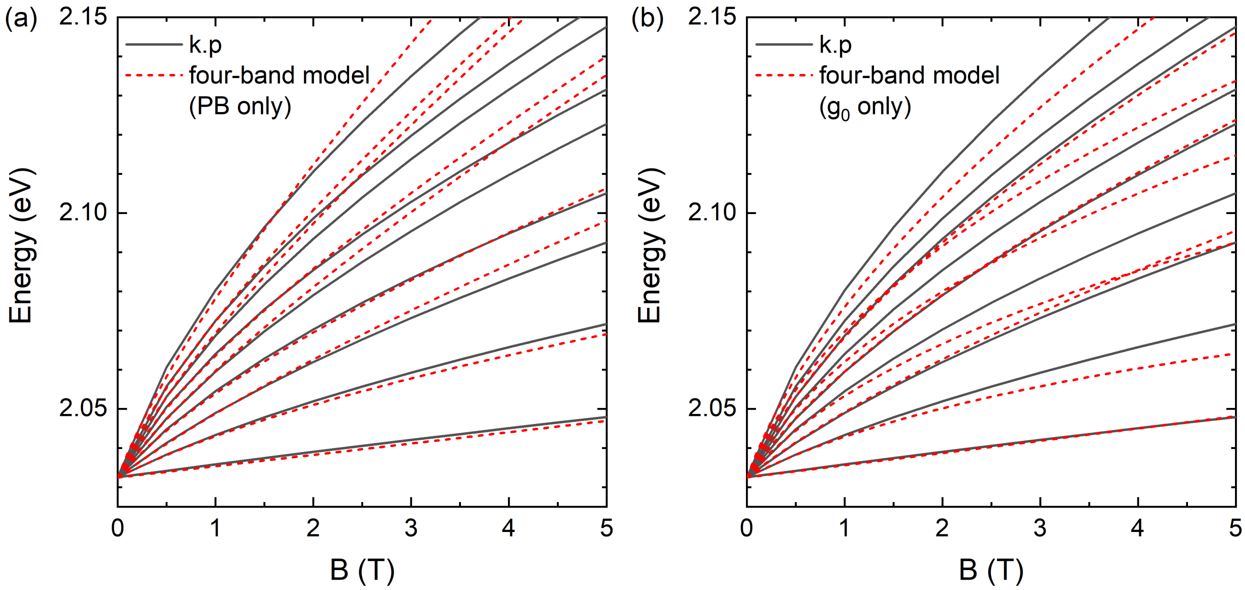

Supplementary Fig. 3. Extended LL fitting between two models. Extended fitting between the $k \cdot p$ model (solid lines) and the four-band model (dash lines) in an InAsSb/InSb SL with a period of 3-nm/1.69-nm. The $k \cdot p$ results in both panels are the same. But in (a), we assume the LL (spin) splitting is due to the PB component M_1 only by setting $g_0 = 0$, while in (b), we consider the opposite case by setting $M_1 = 0$.

described by the g-factor g_0. The parameters used here are Fermi velocity $v_F = 9 \times 10^5$ m/s, $M_1 = 1.9$ eV nm^2 (or equivalently $g_{eff} = 110$), $g_0 = 0$ for Supplementary Fig. 3(a), and $v_F = 7 \times 10^5$ m/s, $M_1 = 0$ eV nm^2, $g_0 = -110$ for Supplementary Fig. 3(b). Even though the Fermi velocities are slightly different in the two cases, we see that at low fields ($B < 1$ T), both mechanisms give equally well fits to the $k \cdot p$ LLs. However, when the magnetic field increases, g_0 fails to produce the correct LL dispersion, while the M_1 contribution still maintains reasonable agreement. From Eq. (3) of the main text, we can see that the main difference between the two models is from the field induced gap term (M_B, which is related to M_1). Therefore, we can conclude that the M_1 parameter is primarily responsible for the (spin) splitting of the LLs in InAsSb/InSb SLs.

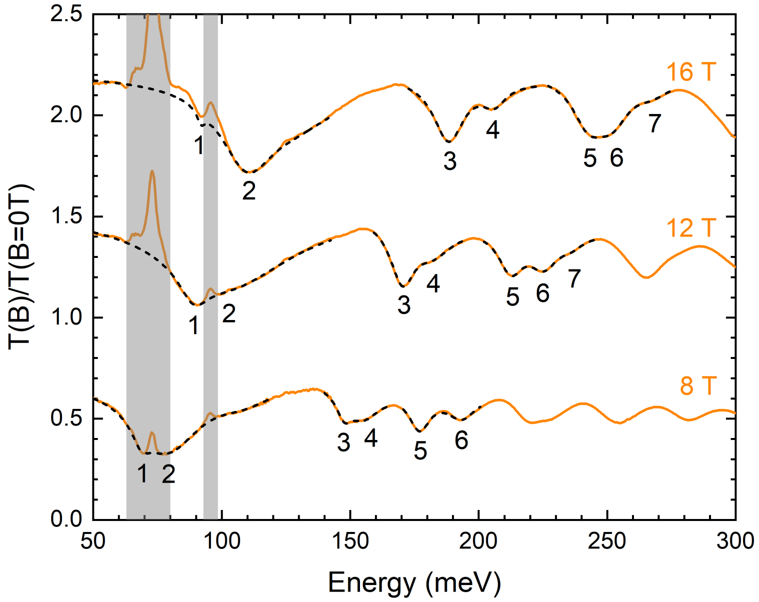

Supplementary Fig. 4. Examples of raw spectra. Normalized transmission spectra, $T(B)/T(0)$, of sample 1445 at selected magnetic fields. Black dash lines are multi-Lorentzian fits to the data, corresponding to LL transitions. Low-lying transitions (or modes) are numbered in the same way as in Fig. 3 of the main text. Gray areas indicate the distorted spectral lineshape due to the presence of two strong modes that do not shift with increasing magnetic field.

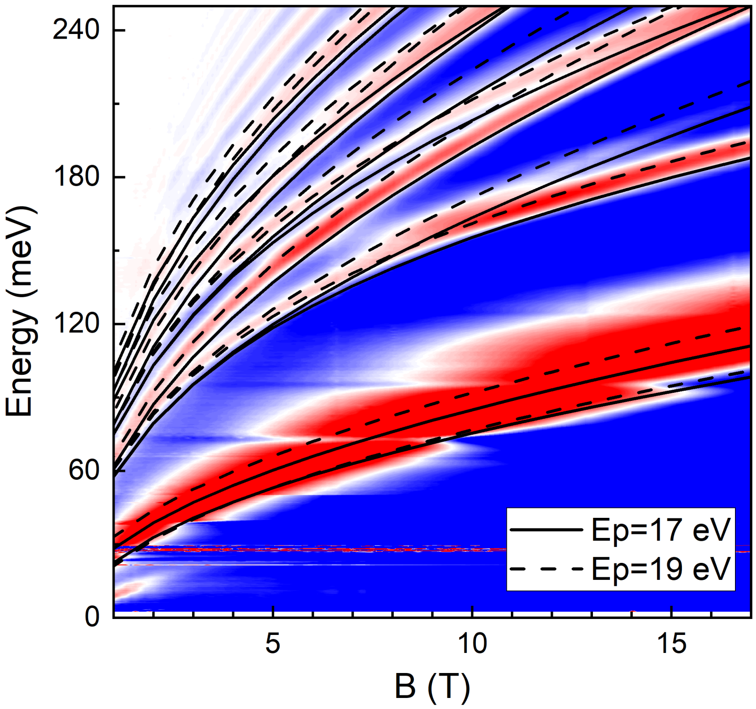

Supplementary Fig. 5. Error estimation in k.p fitting. Calculated LL transition energies for sample 1445 using the k.p model with different E_p values. The calculation results are overlaid on the false color map of the magneto-absorption spectra shown in Fig. 3(a) of the main text.

SUPPLEMENTARY NOTE 5. EFFECTIVE g-FACTOR ERROR ESTIMATION

In this work, we determined the g-factor error bars (Fig. 6(b) of the main text) through the following steps.

First, we extract the LL transition energies from the magneto-absorption spectra. Supplementary Fig. 4 shows a few raw spectra after normalizing to the zero field. The LL transitions can be identified as absorption dips in the spectra with a Lorentzian lineshape. The black dash lines are multi-Lorentzian fits to the data at low energies. The fits capture the spectral lineshape of interband LL transitions well (modes 3, 4, 5 ...), with a typical error as small as ±0.2 meV for each mode. Mode 2 exhibits an unusual broadening. But nevertheless, one can determine its central energy with great accuracy using Lorentzian fitting. The fit to mode 1, which is a CR mode, is affected by the presence of two strong modes at low energies (gray areas) whose energy position is independent of magnetic field. However, once we determine all the interband LL transitions, the energy of this CR mode is known and can be deduced from the LL fan diagram shown in Fig. 3(b,d) of the main text.

Second, we recognize that in our k.p fitting, the main fitting parameter is the Kane energy E_p. When using $E_p = 18$ eV (Fig. 3(a) of the main text), the calculated LL transitions pass the centers of most interband absorption modes and exhibit a good fit. In Supplementary Fig. 5, we examine the robustness of the fitting by manually increasing or decreasing E_p to $E_p = 19$ eV (dash line) or $E_p = 17$ eV (solid line) while keeping all the other parameters of sample 1445 unchanged. We find that both the dash and solid lines are not a good fit to the centers of interband LL transitions but reasonably capture the boundaries (or broadening) of the transitions. Therefore, we conclude that the error bar of E_p for the k.p fitting to the sample 1445 data is ± 1 eV.

Third, given the above error bar, we calculate the corresponding LLs using $E_p = 17$ eV and $E_p = 19$ eV and extract the g-factors in the low field regime ($B \leq 1$ T). This way, we can define the upper and lower bound of the g-factors. The results are shown in Fig. 6(b) of the main text.

SUPPLEMENTARY NOTE 6. ORIGIN OF THE BAND SPLITTING

In this section, we discuss the origin of the M_1 parameter in the four-band model and its connection to the $k \cdot p$ model. Due to the large quantization energy in our SLs, we can follow Ref. [15] and treat the in-plane dispersion as perturbation, that is

$$H(k_x, k_y, k_z) = H_0(k_z) + \Delta H(k_x, k_y).$$

In this way, the wavefunction along the z-direction $|i\rangle_z$ can be determined by considering the eigenvalue problem $H_0 |i\rangle_z = E_i |i\rangle_z$ under proper boundary conditions while the in-plane wavefunction is still plane-wave-like due to its translational symmetry. Here, the eigenvalue E_i corresponds to the band edges of different subbands.

Next, we can construct the four-band effective Hamiltonian H' in the subspace of $|1\rangle$,$|2\rangle$,$|3\rangle$,$|4\rangle$, which corresponds to the conduction and heavy-hole bands with up and down spins. Using degenerate perturbation theory [16], the Hamiltonian H' close to Γ point reads

$$H'_{ij} = \langle i| H_0 |j\rangle + \langle i| \Delta H |j\rangle + \frac{1}{2} \sum_l \langle i| \Delta H |l\rangle \langle l| \Delta H |j\rangle \left[\frac{1}{E_i - E_l} + \frac{1}{E_j - E_l} \right] + ...,$$

where (i, j) and l denote the states inside and outside the subspace, respectively. Then, one can express the M_1 parameter of the ith band as

$$M_1^i \propto \sum_l \frac{| \langle i | \, \Delta H_{il} \, | l \rangle |^2}{E_i - E_l}.$$

This expression involves all the components to understand the LL splitting effect. First, the LL (spin) splitting is due to the interactions with other bands outside the subspace. It is consistent with the expectation that the LLs are strictly spin-degenerate in a pure four-band model, and any splitting ought to come from contributions of additional bands. Second, the M_1^i is related to the sum of the matrix elements $\langle i | \, \Delta H_{il} \, | l \rangle$ between different bands. Therefore, the wavefunction overlap and mixing can inevitably change the values of the matrix elements and are responsible for the g-factor engineering in our system.

---

[1] Y. Jiang, *Magneto-infrared spectroscopy of emerging topological materials*, Ph.D. thesis, Georgia Institute of Technology (2017).

[2] Y. Jiang, S. Thapa, G. Sanders, C. Stanton, Q. Zhang, J. Kono, W. Lou, K. Chang, S. Hawkins, J. Klem, *et al.*, Physical Review B **95**, 045116 (2017).

[3] G. D. Sanders, Y. Sun, F. V. Kyrychenko, C. J. Stanton, G. A. Khodaparast, M. A. Zudov, J. Kono, Y. H. Matsuda, N. Miura, and H. Munekata, Physical Review B **68**, 165205 (2003).

[4] J. Li, W. Yang, and K. Chang, Physical Review B **80**, 035303 (2009).

[5] R. Wood, D. Saha, L. McCarthy, J. Tokarski III, G. Sanders, P. Kuhns, S. McGill, A. Reyes, J. Reno, C. Stanton, *et al.*, Physical Review B **90**, 155317 (2014).

[6] I. Vurgaftman, J. R. Meyer, and L. R. Ram-Mohan, Journal of Applied Physics **89**, 5815 (2001).

[7] S. N. Smith, C. C. Phillips, R. H. Thomas, R. A. Stradling, I. T. Ferguson, A. G. Norman, B. N. Murdin, and C. R. Pidgeon, Semiconductor Science and Technology **7**, 900 (1992).

[8] P. T. Webster, N. A. Riordan, S. Liu, E. H. Steenbergen, R. A. Synowicki, Y.-H. Zhang, and S. R. Johnson, Journal of Applied Physics **118**, 245706 (2015).

[9] B. A. Foreman, Physical Review B **56**, R12748 (1997).

[10] O. Berolo and J. C. Woolley, in *Proceeding of the 11th International Conference on the Physics of Semiconductors (ICPS)* (Warsaw, 1972) p. 1420.

[11] S. Suchalkin, J. Ludwig, G. Belenky, B. Laikhtman, G. Kipshidze, Y. Lin, L. Shterengas, D. Smirnov, S. Luryi, W. L. Sarney, and S. P. Svensson, Journal of Physics D: Applied Physics **49**, 105101 (2016).

[12] S. A. Cripps, T. J. C. Hosea, A. Krier, V. Smirnov, P. J. Batty, Q. D. Zhuang, H. H. Lin, P.-W. Liu, and G. Tsai, Applied Physics Letters **90**, 172106 (2007).

[13] J. E. Sestoft, T. Kanne, A. N. Gejl, M. von Soosten, J. S. Yodh, D. Sherman, B. Tarasinski, M. Wimmer, E. Johnson, M. Deng, J. Nygård, T. S. Jespersen, C. M. Marcus, and P. Krogstrup, Phys. Rev. Materials **2**, 044202 (2018).

[14] C. M. Moehle, C. T. Ke, Q. Wang, C. Thomas, D. Xiao, S. Karwal, M. Lodari, V. van de Kerkhof, R. Termaat, G. C. Gardner, G. Scappucci, M. J. Manfra, and S. Goswami, Nano Letters **21**, 9990 (2021).

[15] B. Laikhtman, S. Suchalkin, and G. Belenky, Physical Review B **95**, 235401 (2017).

[16] C.-X. Liu, X.-L. Qi, H. Zhang, X. Dai, Z. Fang, and S.-C. Zhang, Physical Review B **82**, 045122 (2010).
